# Restricted TcR β chain CDR3 clonotype is associated with resolved acute hepatitis B subjects

**DOI:** 10.1186/s12879-021-05816-2

**Published:** 2021-01-23

**Authors:** Dangsheng Xiao, Ju Wang, Zhitao Chen, Xiuyuan Jin, Yirui Xie, Dong Yan, Jiezuan Yang

**Affiliations:** 1 Zhejiang Provincial Key Laboratory for Diagnosis and Treatment of Aging and Physic-chemical Injury Diseases, Department of Geriatrics, the First Affiliated Hospital, Zhejiang University School of Medicine, Zhejiang, 310003 Hangzhou China; 2 State Key Laboratory for Diagnosis and Treatment of Infectious Diseases, National Clinical Research Center for Infectious Diseases, Collaborative Innovation Center for Diagnosis and Treatment of Infectious Diseases, the First Affiliated Hospital, Zhejiang University School of Medicine, Zhejiang, 310003 Hangzhou China; 3 Department of Hepatobiliary and Pancreatic Surgery, the First Affiliated Hospital, Zhejiang University School of Medicine, Zhejiang, 310003 Hangzhou China

**Keywords:** T cell receptor, Complementarity-determining region 3, Resolved acute hepatitis B, HBsAg seroconversion, High throughput sequencing

## Abstract

**Background:**

T cells play an important role in the prognosis of hepatitis B virus (HBV) infection, and are involved in the seroconversion of a patient from HBsAb negative to positive. To compare the T-cell receptor β-chain variable region (TcRBV) complementarity-determining region 3 (CDR3) in subjects with or without hepatitis B surface antigen (HBsAg) convert to hepatitis B surface antibody (HBsAb), the TcRBV was determined using high throughput sequencing (HTS).

**Methods:**

The clonotype and diversity of CDR3 in peripheral blood mononuclear cells of subjects with resolved acute hepatitis B (AHB, HBsAb+, HBsAg-) (*n* = 5), chronic hepatitis B (CHB, HBsAb-, HBsAg+) (n = 5), and healthy controls (HC, HBsAb-, HBsAg-) (*n* = 3) were determined and analyzed using HTS (MiSeq).

**Results:**

The overlapping rate of CDR3 clones of any two samples in AHB group was 2.00% (1.74% ~ 2.30%), CHB group was 1.77% (1.43% ~ 2.61%), and HC group was 1.82% (1.62% ~ 2.12%), and there was no significant difference among the three groups by Kruskal-Wallis H test. However, among the top 10 cumulative frequencies of clonotypes, only the frequency of clonotype (TcRBV20–1/BD1/BJ1–2) in AHB group was lower than that of HC group (*P* < 0.001). Moreover, exclude the 10 top clonotypes, there are 57 markedly different frequency of clones between AHB and CHB groups (18 clones up, 39 clones down), 179 (180–1) different clones between AHB and HC groups, and 134 different clones between CHB and HC groups. With regard to BV and BJ genotypes, there was no significant different frequency among the groups. Furthermore, there was no significant difference in the diversity of TcRBV CDR3 among the three groups (*P* > 0.05).

**Conclusions:**

Thus, there are 57 TcRBV clonotypes that may be related to HBsAg seroconversion of AHB subjects, but the diversity of TcRBV CDR3 is not significantly related to the HBsAb positive status.

**Supplementary Information:**

The online version contains supplementary material available at 10.1186/s12879-021-05816-2.

## Background

Hepatitis B virus (HBV) can cause acute and chronic HBV infection, liver cirrhosis, and liver cancer, and is a serious threat to human health worldwide [1]. In recent years, with the wide spread of hepatitis B vaccine immunization and the application of antiviral medicine, acute hepatitis B infection has been significantly reduced, and chronic infection has been effectively controlled [2]. The appearance of HBsAb (HBsAg seroconversion) in peripheral blood indicates that the acute hepatitis B subject has recovered with a protective effect on body from recurrent HBV infection. HBsAg seroconversion (SC) is also an ideal prognosis (favourable outcome) for CHB patients who are undergoing antiviral treatment [3, 4].

**Fig. 1:**
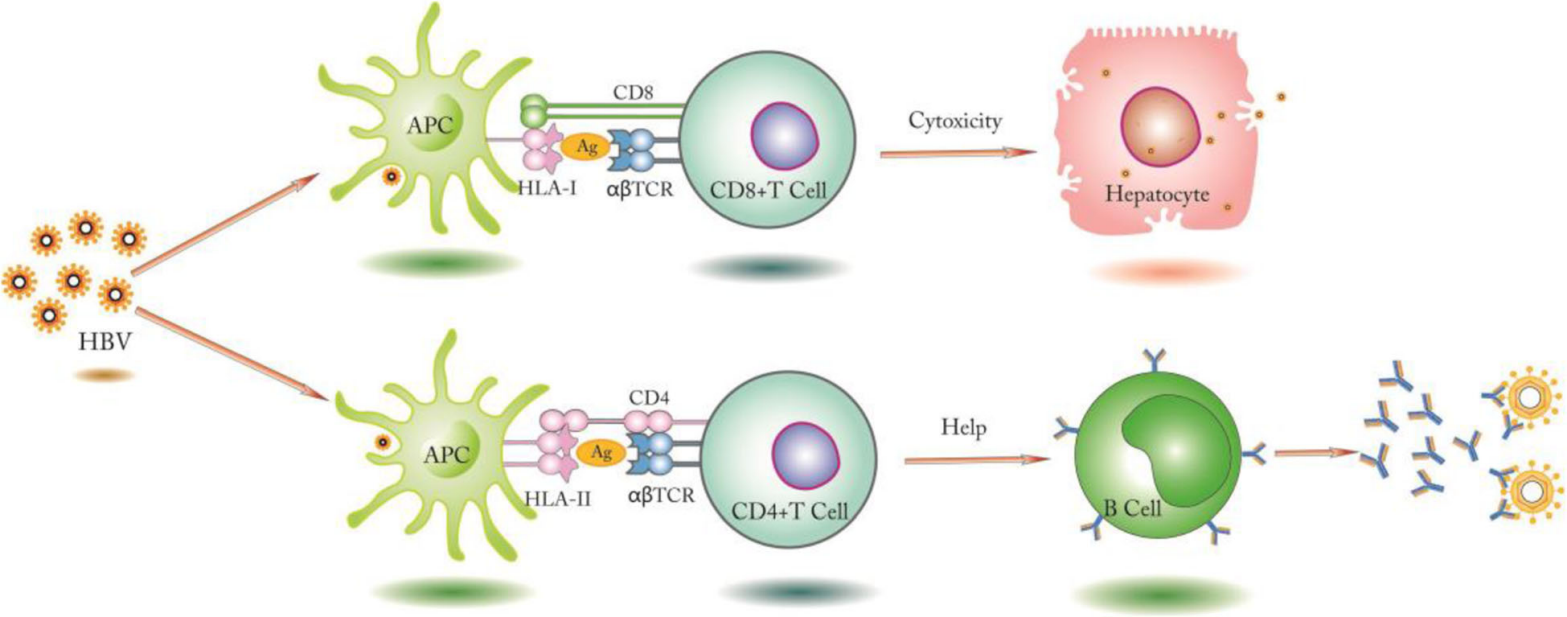
HBV infection and related immune response. HBV enters hepatocytes or phagocytes, and CD8+/ CD4+ T cells recognize HBV antigens presented by MHC I or MHC II, then CD8+ T cells are activated and cytotoxic effect on target cells, and the CD4+ T cells help B cells activate and produce antibodies against HBV

At present, most of studies on HBsAg SC are based on the virus level of HBV itself, but the exact mechanism of the production of HBsAb (resulting from HBsAg SC) is still unclear [3]. It is generally believed that host would produce antigen-specific T cells whose function is determined by the T cell receptor (TcR) on its surface and these T cell could help B cells to produce HBsAb, when a pathogen or a foreign antigen invades a host [5, 6]. Additionally, the simple description of route HBV transmission is following, hepatocytes are the host cell of HBV. By the interaction between antigens on HBV envelope and the receptors of the hepatocytes, HBV virion is endocytosized and encapsulated as an endosomal vesicle in hepatocytes. More details are shown in our previous report [7]. Moreover, the HBV antigen is presented to T cells by MHC I (MHC II) on the membrane of presenting cell, and development of cell-mediated and humoral immunity against HBV (Fig. 1). However, HBV infection would be persistent if the T cells are not activated effectively and rendered anergy.

T cell receptor (TcR) is a receptor molecule on the T-cell membrane that recognizes the antigen. TcR is a heterodimer composed of α, β chains or γ, δ chains, and more than 95% of mature T-cells in human peripheral blood present the α and β chains. TcR is the key element for the T cell to activate, recognize, and combine with the peptide/MHC complex [8, 9]. The complexity of the TcR structure is mainly determined by the complementary decision region 3 (CDR3) that is encoded by variable (V), diversity (D), and joint (J) and their reorganization and editing. In the process of lymphocyte maturation, different CDR3 genotypes are formed by the rearrangement, insertion, mutation and deletion of V, D, and J. Different genotypes indicate different nucleotide sequences, giving rise to amino acid sequences translated in the ribosome, and forming a variety of CDR3 clones [10, 11]. Therefore, by measuring the diversity of a specific TcR CDR3 sequence and the frequency change in different physiological or pathological states, we can clearly understand the extent of clonal expansion of corresponding T lymphocytes, and understand the role of T cells in HBsAg seroconversion.

In the present study, TcRBV CDR3 clones of resolved acute hepatitis B (AHB) (HBsAb+) and CHB (HBsAb-) were analyzed to explore the different profiles of CDR3 expression in AHB subjects with HBsAb+ after infection with HBV, to clarify the role of T cell subsets in the mechanism of HBsAg SC.

## Methods

### Subjects enrolled

Between January and May 2019, five subjects with resolved acute hepatitis B (AHB) and five chronic hepatitis B (CHB) subjects were enrolled in our study. All subjects were selected at the health examination center of the First Affiliated Hospital, Zhejiang University School of Medicine.

Our diagnostic criteria for AHB and CHB met the Guidelines for the prevention and treatment of chronic hepatitis B (version 2015) and the diagnostic criteria described in other reports [12]. In brief, the CHB subjects are defined as those who are with HBsAg-positive (> 6 months), HBeAg-positive and HBV DNA detectable, and with abnormal liver function tests. The AHB subjects are collected based on normal biochemical indexes of liver function, and had transient mild symptoms of HBV infection in the past, at present, HBsAb-positive and HBcAb-positive without any clinical symptoms, absence of HBsAg, and HBV DNA levels below the detection limit, without a history of hepatitis B vaccination. Additionally, the exclusion criteria for AHB and CHB were described in our previous report [10]. Three healthy controls were negative for all HBV serological markers and displayed no clinical or laboratory evidence of other infectious diseases or immunological disorders. Additional characteristics of enrolled subjects at the time of the study are shown in Table 1.

**Table 1:** Demographic and clinical characteristics of the enrolled subjects

Characteristics	AHB	CHB	HC
No. subject	5	5	3
Gender (male/female)	5/0	4/1	1/2
Mean age (years)	32.3 ± 7.9	43.3 ± 12.3	34.3 ± 9.9
ALT level (U/L)	14.0 (10.0–30.0)	94.5 ± 37.7	23 ± 8.2
TBi (umol/L)	11.0 ± 5.3	13.0 (9.5–13.4)	7.9 (6.4–16.5)
HBV DNA (log10, Copies/mL)	UD	7.5 ± 1.5	UD
HBV genotype	UD	2B/3C	UD
HBeAg (PEIU/mL)	0.38 (0.07–0.40)	218.67(0.30–459.54)	0.25 ± 0.16
HBcAb (S/CO)	3.13 (2.09–5.73)	8.77 ± 0.68	0.10 ± 0.02
HBsAg (log10 IU/mL)	UD	4.0 ± 0.8	UD
HBsAb (mIU/mL)	626.9 (358.1–670.9)	0.4 (0.2–0.9)	0.0 (0.0–0.135)

All subjects are of Chinese Han ethnicity. Values are expressed as median (Q1-Q3), normal distribution data expressed as mean ± SD (standard deviation), unless otherwise indicated

*UD* undetectable

Normal values: ALT ≤40 U/L; TBi ≤21 umol/L

This project and protocols involving human subjects were approved by the ethics committee of the First Affiliated Hospital, Zhejiang University School of Medicine. Informed consent was obtained from each subject enrolled in the study. The study protocol meets the ethical principles of the Declaration of Helsinki (2008).

### Biochemical, serological, and HBV DNA load assay

Biochemical indexes of liver function were detected by an automatic biochemical analyzer (HITACHI 7600, Japan) at clinical laboratory of our unit. Plasma samples were used to detect hepatitis B serological indicators (such as, HBsAg, HBsAb, HBcAb, HBeAg, and HBeAb) with enzyme immunoassays (Abbott, Chicago, IL, USA) according to the test guidance. The HBV DNA level was quantitatively determined using real-time polymerase chain reaction (qPCR) [13] with a commercial kit, and the detection limit is 30 copies/ml. Briefly, 5 μL of serum was added to the reaction mixture containing 38 μL of HBV PCR mixture and 2 μL of enzyme mixture. Quantification of HBV DNA was performed using a qPCR thermocycler (Applied Biosystem 7300, Foster City, California, USA). HBV genotypes were determined with sequence detection via PCR, the detail was shown in our previous report [14].

### PBMC separation and RNA extraction

Peripheral blood mononuclear cells (PBMCs) were isolated from peripheral venous whole blood samples using Ficoll (Haoyang, Tianjin, China) density gradient centrifugation. Immediately, total RNA was extracted from PBMCs using Trizol (Invitrogen, Carlsbad, CA, USA) following the operating instructions, the more detail following our previously report [10]. Finally, the quality of RNA was evaluated using an Agilent 2100 Bioanalyzer (Agilent Technologies, USA).

### Construction of sequencing library and TcRBV sequencing

The full-length TcRBV gene was amplified by 5′ RACE unbiased amplification protocol, and constructed the sequencing libraries. Concentration of the TcRBV library, integrity of the fragment, size of the inserted fragment, and effective concentration of the library were detected and quantified using Qubit 2.0 Fluorometer, Agilent 2100 and Q-PCR respectively. Subsequently, the TcRBV library was sequenced using the ImmuHub™ *TCR* profiling system at the deep level (ImmuQuad Biotech, Hangzhou, China). The raw sequence was screened, the low-quality sequences with splices were removed, the sequencing background was filtered [15], and the sequence obtained was compared with the reference sequences of the international ImMunoGeneTics (IMGT) database (www.imgt.org). The nucleotide and amino acid (AA) sequences of TcR β chain complementarity determining region 3 (CDR3) were determined, and those with out-of-frame and stop codon sequences were removed from the identified TcR β chain repertoire as per more detailed steps described in the previous report [16, 17].

### CDR3 diversity analysis

The diversity of CDR3 sequence was analyzed and presented using Pielous / diversity, Shannon entropy index (SE), and Inverse Simpson’s index (IS), which have been widely used for assessing the richness and diversity of TcR data [18–20]. The formula is as follows:

$$ \mathrm{SE}=\hbox{-} {\sum}_{i=1}^s Pi\ln Pi $$

*P*i is the proportion of characters belonging to the i^th^ clone type of i in the string, S is the total number of TcRBV.

$$ \mathrm{IS}=1/{\sum}_{i=1}^s{Pi}^2 $$

*P*i is the proportion of characters belonging to the i^th^ clone type of i in the string, S is the total number of TcRBV. To determine the usage of each V/D/J gene in the TcRBV repertoire, the number of each V/D/J gene assigned to different TcRBV clonotype was summed.

Baroni-Urbani and Buser (BUB) overlap index [21] was calculated according to respective formulae, and the more detailed method was described previously [16].

### Statistical analysis

The statistical programming language R (version 2.8.1) and GraphPad Prism 7.0 Package (San Diego, CA, USA) were used to analyze the experimental results. The mean ± standard deviation (SD) was used to describe the data of the normal distribution, and the data for the skewed distribution was described by the median (25 quantiles, 75 quantiles). The Kruskal-Wallis H test was used for comparing the SE and IS ratio, clonal overlapping rate, and the frequency of dominant genotypes among the three groups. Mann-Whitney U test was used to compare two groups among the three groups. The frequencies of the clone and V, J genotypes were compared by independent sample T-tests. Comparisons between groups were performed using two-tailed T-tests. *P* values < 0.05 were considered statistically significant.

## Results

### TcRBV CDR3 sequence immune repertoire (IR)

The original sequences obtained by high-throughput sequencing (HTS) were converted to raw reads by filtering the low-quality sequences such as by removing the sequence with length less than 150 bp, the spliced sequences, and cutting out the continuous low-quality bases with a mass value less than 19 at both ends of the sequence. The sequence with undetermined base proportion more than 10% was also removed, so that more than 80% of the filtered sequence had a sequencing quality value (Pherd value) of more than 30. Additionally, V, J, and CDR3 regions of TcRBV consensus sequences were identified using BLAST Plus in the IMGT information system using a standard algorithm [22].

### Sample amplification and diversity

There was no difference in the number of reads between the three groups (*P* > 0.05), and in the number of CDR3 or in the unique CDR3 (both, P > 0.05). However, the ratio of unique CDR3/CDR3 in the CHB group was significantly lower than that in the HC group (*P* = 0.0348), but no significant difference was found between the AHB and HC groups (Additional file 1: Tab. S1). Additionally, the diversity index was used to represent the diversity of each group, and no significant difference was found among these groups.

### Frequency of V/D/J combination compared between groups

All V/D/J combinations of each sample were obtained by IMGT database comparisons, and the top 10 V/D/J gene combinations with the highest frequency from 13 samples were selected (Table 2). There was no significant difference in the frequency of the top 10 V/D/J gene combinations among the three groups, except the V/D/J (TcRBV20–1/BD1/BJ1–2) between AHB and HC group (average frequency, 6.457E-6 vs. 8.310E-6, *P* = 6.434E-3).

**Table 2:** Top ten cumulative frequencies of V/D/J gene combinations

V/D/J	Frequency
TRBV24–1/TRBD2/TRBJ2–1	0.468146
TRBV5–1/TRBD1/TRBJ1–2	0.142069
TRBV20–1/TRBD1/TRBJ1–2^a^	0.130259
TRBV9/TRBD1/TRBJ1–1	0.103324
TRBV25–1/TRBD1/TRBJ2–5	0.092293
TRBV7–8/TRBD2/TRBJ2–7	0.072890
TRBV4–2/ /TRBJ2–1	0.058674
TRBV14/TRBD1/TRBJ1–1	0.050959
TRBV7–6/TRBD2/TRBJ2–1	0.049602
TRBV12–4/TRBD1/TRBJ2–4	0.043804

^a^ There is significantly difference between AHB and HC groups

Furthermore, the remaining (excluding the top ten) frequencies were compared between the three groups, the clone genotype TcRBV6–4/BD1 (BD2) /BJ2–2 in the AHB group was 1.366E-05 (average frequency), and the frequency of the CHB group was 8.851E-06, the difference between the two groups was statistically significant (*P* = 0.0021). Simultaneously, the clone genotype TcRBV6–4/BD1 (BD2) /BJ2–2 in the HC group was 1.068E-05, the difference between the AHB and HC groups was also statistically significant (*P* = 0.0407). Furthermore, there were 57 different clones between the AHB and CHB groups, 180 different clones between the AHB and HC groups, and 135 different clones between the CHB and HC groups with significant difference (Tables 3, 4 and 5; Additional file 2: Fig. S1, S2).

**Table 3:** Comparison of clonal frequency (V-J) between AHB and CHB (P < 0.05)

VJ	P	Mean value -comparing	VJ	P	Mean value -comparing
TRBV27|TRBJ1–4	0.00017	AHB < CHB	TRBV6–5|TRBJ1–1	0.01937	AHB < CHB
TRBV7–2|TRBJ2–3	0.00035	AHB < CHB	TRBV4–1|TRBJ1–4	0.01939	AHB < CHB
TRBV7–2|TRBJ2–2	0.00041	AHB < CHB	TRBV7–2|TRBJ2–6	0.01990	AHB < CHB
TRBV10–3|TRBJ1–4	0.00042	AHB < CHB	TRBV7–2|TRBJ1–4	0.02215	AHB < CHB
TRBV6–2|TRBJ2–4	0.00059	AHB < CHB	TRBV5–1|TRBJ1–6	0.02320	AHB < CHB
TRBV4–2|TRBJ1–2	0.00156	AHB < CHB	TRBV5–5|TRBJ1–5	0.02323	AHB < CHB
TRBV6–4|TRBJ2–2	0.00208	AHB > CHB	TRBV28|TRBJ1–3	0.02407	AHB > CHB
TRBV5–6|TRBJ1–2	0.00299	AHB < CHB	TRBV13|TRBJ2–5	0.02524	AHB < CHB
TRBV4–1|TRBJ2–4	0.00319	AHB < CHB	TRBV6–2|TRBJ2–7	0.02594	AHB < CHB
TRBV5–6|TRBJ1–6	0.00432	AHB < CHB	TRBV11–3|TRBJ2–1	0.02712	AHB > CHB
TRBV20–1|TRBJ2–5	0.00493	AHB > CHB	TRBV12–3|TRBJ1–6	0.02727	AHB < CHB
TRBV6–1|TRBJ2–3	0.00521	AHB > CHB	TRBV11–3|TRBJ1–1	0.02987	AHB > CHB
TRBV11–3|TRBJ2–2	0.00598	AHB > CHB	TRBV5–6|TRBJ2–4	0.02996	AHB < CHB
TRBV14|TRBJ1–6	0.00607	AHB > CHB	TRBV5–4|TRBJ2–7	0.03061	AHB > CHB
TRBV7–7|TRBJ2–5	0.00631	AHB < CHB	TRBV6–6|TRBJ1–2	0.03276	AHB < CHB
TRBV7–2|TRBJ2–4	0.00737	AHB < CHB	TRBV29–1|TRBJ2–7	0.03291	AHB > CHB
TRBV11–3|TRBJ2–4	0.00748	AHB > CHB	TRBV21–1|TRBJ1–6	0.03520	AHB < CHB
TRBV11–2|TRBJ1–3	0.00774	AHB < CHB	TRBV6–4|TRBJ2–6	0.03847	AHB > CHB
TRBV18|TRBJ1–4	0.00793	AHB < CHB	TRBV7–6|TRBJ1–6	0.04088	AHB < CHB
TRBV30|TRBJ1–4	0.00802	AHB > CHB	TRBV7–6|TRBJ1–2	0.04124	AHB < CHB
TRBV10–3|TRBJ1–6	0.00852	AHB < CHB	TRBV7–8|TRBJ1–6	0.04200	AHB < CHB
TRBV30|TRBJ1–3	0.01086	AHB > CHB	TRBV15|TRBJ1–5	0.04326	AHB < CHB
TRBV5–8|TRBJ1–3	0.01107	AHB > CHB	TRBV2|TRBJ1–1	0.04336	AHB < CHB
TRBV10–2|TRBJ1–6	0.01131	AHB < CHB	TRBV10–3|TRBJ1–5	0.04544	AHB < CHB
TRBV11–1|TRBJ1–2	0.01266	AHB < CHB	TRBV27|TRBJ1–3	0.04720	AHB > CHB
TRBV7–2|TRBJ2–1	0.01369	AHB < CHB	TRBV3–1|TRBJ2–7	0.04920	AHB < CHB
TRBV4–2|TRBJ1–5	0.01422	AHB < CHB	TRBV7–3|TRBJ1–2	0.04964	AHB < CHB
TRBV10–1|TRBJ1–3	0.01847	AHB > CHB	TRBV10–2|TRBJ1–5	0.04972	AHB < CHB
TRBV12–3|TRBJ2–2	0.01877	AHB > CHB

**Table 4:** Comparison of clonal frequency (V-J) between AHB and HC (P < 0.001)

VJ	P	Mean value -comparing	VJ	P	Mean value -comparing
TRBV28|TRBJ1–2	4.80E-24	AHB < HC	TRBV11–2|TRBJ1–2	2.34E-05	AHB < HC
TRBV7–2|TRBJ1–6	4.02E-18	AHB < HC	TRBV29–1|TRBJ1–5	3.30E-05	AHB < HC
TRBV5–4|TRBJ2–3	3.46E-15	AHB < HC	TRBV12–3|TRBJ1–1	3.39E-05	AHB < HC
TRBV18|TRBJ2–1	2.54E-13	AHB < HC	TRBV18|TRBJ1–1	3.78E-05	AHB < HC
TRBV6–2|TRBJ2–1	2.59E-12	AHB < HC	TRBV4–1|TRBJ2–4	7.19E-05	AHB < HC
TRBV28|TRBJ2–1	7.71E-11	AHB < HC	TRBV15|TRBJ2–1	8.21E-05	AHB < HC
TRBV7–2|TRBJ2–6	1.61E-10	AHB < HC	TRBV2|TRBJ1–1	9.10E-05	AHB < HC
TRBV18|TRBJ2–2	5.65E-10	AHB < HC	TRBV10–3|TRBJ1–1	9.46E-05	AHB < HC
TRBV11–2|TRBJ1–3	9.66E-10	AHB < HC	TRBV19|TRBJ1–2	0.000113473	AHB < HC
TRBV6–2|TRBJ2–4	2.93E-09	AHB < HC	TRBV19|TRBJ1–5	0.000117244	AHB < HC
TRBV18|TRBJ1–5	5.11E-09	AHB < HC	TRBV5–4|TRBJ2–6	0.000118196	AHB < HC
TRBV5–5|TRBJ2–5	6.09E-09	AHB < HC	TRBV19|TRBJ1–6	0.000142831	AHB < HC
TRBV20–1|TRBJ1–2	6.43E-09	AHB < HC	TRBV9|TRBJ1–3	0.000190044	AHB < HC
TRBV28|TRBJ1–3	1.56E-08	AHB < HC	TRBV5–5|TRBJ1–5	0.000199098	AHB < HC
TRBV18|TRBJ1–4	2.45E-08	AHB < HC	TRBV3–1|TRBJ2–7	0.000218947	AHB < HC
TRBV7–2|TRBJ2–3	2.69E-08	AHB < HC	TRBV6–1|TRBJ1–3	0.000227591	AHB < HC
TRBV7–2|TRBJ2–4	3.04E-08	AHB < HC	TRBV28|TRBJ1–1	0.000285611	AHB < HC
TRBV7–2|TRBJ1–1	4.53E-08	AHB < HC	TRBV5–4|TRBJ2–2	0.000286058	AHB < HC
TRBV23–1|TRBJ2–3	9.69E-08	AHB < HC	TRBV5–4|TRBJ1–2	0.000306129	AHB < HC
TRBV5–5|TRBJ2–1	1.12E-07	AHB < HC	TRBV12–3|TRBJ1–2	0.000315338	AHB < HC
TRBV6–2|TRBJ2–7	1.47E-07	AHB < HC	TRBV11–2|TRBJ2–2	0.000315564	AHB < HC
TRBV20–1|TRBJ2–3	1.83E-07	AHB < HC	TRBV10–3|TRBJ1–6	0.000323084	AHB < HC
TRBV5–6|TRBJ2–5	2.00E-07	AHB < HC	TRBV14|TRBJ2–7	0.000435467	AHB < HC
TRBV23–1|TRBJ2–5	2.59E-07	AHB < HC	TRBV23–1|TRBJ2–1	0.000520227	AHB < HC
TRBV7–3|TRBJ2–1	3.62E-07	AHB < HC	TRBV6–2|TRBJ2–5	0.000527129	AHB < HC
TRBV12–3|TRBJ2–1	4.29E-07	AHB < HC	TRBV6–5|TRBJ1–2	0.000540139	AHB < HC
TRBV10–3|TRBJ2–5	1.14E-06	AHB < HC	TRBV5–4|TRBJ1–6	0.000558084	AHB < HC
TRBV7–2|TRBJ1–4	1.69E-06	AHB < HC	TRBV18|TRBJ2–6	0.000563219	AHB < HC
TRBV15|TRBJ1–5	4.34E-06	AHB < HC	TRBV6–2|TRBJ2–6	0.000656795	AHB < HC
TRBV11–2|TRBJ2–4	4.37E-06	AHB < HC	TRBV12–5|TRBJ2–1	0.000660101	AHB < HC
TRBV11–1|TRBJ2–1	5.25E-06	AHB < HC	TRBV2|TRBJ1–3	0.000696902	AHB < HC
TRBV5–5|TRBJ1–2	6.84E-06	AHB < HC	TRBV6–5|TRBJ1–5	0.00076708	AHB < HC
TRBV6–2|TRBJ2–2	1.13E-05	AHB < HC	TRBV19|TRBJ1–4	0.000812351	AHB < HC
TRBV10–3|TRBJ1–5	1.41E-05	AHB < HC	TRBV5–8|TRBJ2–3	0.00083316	AHB < HC
TRBV6–5|TRBJ2–4	1.45E-05	AHB < HC	TRBV3–1|TRBJ1–3	0.000874836	AHB < HC
TRBV7–2|TRBJ2–1	1.65E-05	AHB < HC	TRBV4–2|TRBJ2–4	0.000879734	AHB < HC
TRBV28|TRBJ2–7	1.65E-05	AHB < HC	TRBV5–4|TRBJ2–4	0.000890673	AHB < HC

**Table 5:** Comparison of clonal frequency (V-J) between CHB and HC (P < 0.001)

VJ	P	Mean value -comparing	VJ	P	Mean value -comparing
TRBV28|TRBJ1–3	4.67E-17	CHB < HC	TRBV30|TRBJ2–3	2.85E-05	CHB < HC
TRBV5–4|TRBJ2–7	5.96E-17	CHB < HC	TRBV11–1|TRBJ2–1	4.26E-05	CHB < HC
TRBV4–3|TRBJ2–3	6.27E-14	CHB < HC	TRBV27|TRBJ1–3	6.34E-05	CHB < HC
TRBV5–4|TRBJ2–3	9.65E-14	CHB < HC	TRBV11–2|TRBJ2–3	6.67E-05	CHB < HC
TRBV18|TRBJ2–3	1.01E-12	CHB < HC	TRBV6–2|TRBJ2–3	6.72E-05	CHB < HC
TRBV6–2|TRBJ2–1	1.58E-12	CHB < HC	TRBV4–3|TRBJ1–6	8.35E-05	CHB < HC
TRBV28|TRBJ1–4	2.13E-12	CHB < HC	TRBV29–1|TRBJ2–3	8.71E-05	CHB < HC
TRBV19|TRBJ2–4	2.05E-10	CHB < HC	TRBV29–1|TRBJ2–7	0.000146	CHB < HC
TRBV28|TRBJ2–2	2.78E-10	CHB < HC	TRBV6–2|TRBJ2–2	0.000150	CHB < HC
TRBV19|TRBJ2–7	7.28E-10	CHB < HC	TRBV28|TRBJ1–6	0.000159	CHB < HC
TRBV30|TRBJ1–5	4.23E-09	CHB < HC	TRBV7–3|TRBJ2–7	0.000164	CHB < HC
TRBV19|TRBJ1–2	2.03E-08	CHB < HC	TRBV24–1|TRBJ2–7	0.000171	CHB < HC
TRBV19|TRBJ2–3	2.46E-08	CHB < HC	TRBV23–1|TRBJ2–3	0.000181	CHB < HC
TRBV18|TRBJ2–1	2.79E-08	CHB < HC	TRBV20–1|TRBJ2–2	0.000217	CHB < HC
TRBV28|TRBJ2–1	8.28E-08	CHB < HC	TRBV7–2|TRBJ2–6	0.000308	CHB < HC
TRBV4–3|TRBJ2–1	1.05E-07	CHB < HC	TRBV7–3|TRBJ2–5	0.000338	CHB < HC
TRBV4–3|TRBJ1–5	3.68E-07	CHB < HC	TRBV14|TRBJ2–1	0.000503	CHB < HC
TRBV4–3|TRBJ2–2	1.10E-06	CHB < HC	TRBV7–2|TRBJ1–3	0.000532	CHB < HC
TRBV12–5|TRBJ2–1	1.31E-06	CHB < HC	TRBV7–2|TRBJ1–5	0.000657	CHB < HC
TRBV10–3|TRBJ2–7	2.17E-06	CHB < HC	TRBV21–1|TRBJ2–1	0.000718	CHB < HC
TRBV7–3|TRBJ2–1	5.80E-06	CHB < HC	TRBV7–2|TRBJ2–3	0.000784	CHB < HC
TRBV20–1|TRBJ2–5	9.01E-06	CHB < HC	TRBV21–1|TRBJ2–7	0.000882	CHB < HC
TRBV6–2|TRBJ2–7	1.28E-05	CHB < HC			

### Frequency of TcRBV (BJ) compared between groups

**Fig. 2:**
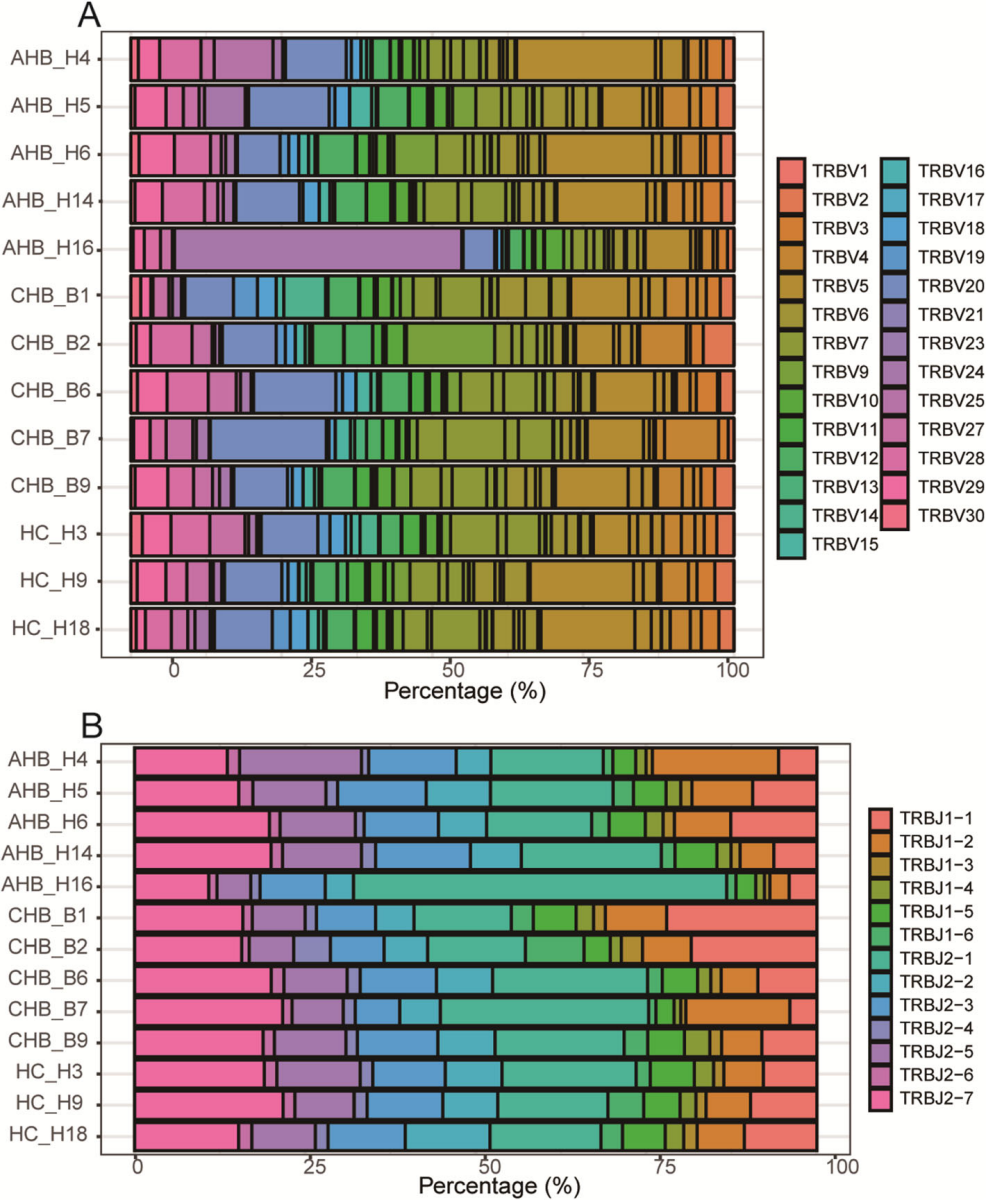
Semi-quantitative results of BV or BJ segment usage in each subject of the three groups (AHB, CHB, and HC). **a** BV segment usage in PBMC from each subject; **b** BJ segment usage in PBMC from each subject

We identified 48 BV and 13 BJ segments, of which 48 BV segments were merged into 27 segments. These results were used to analyze the BV and BJ segment usage (percentage) in each sample, and no significant difference was found for each sample (both, *P* > 0.05) (Fig. 2) except BV24 and BJ2–1 of H16 sample. The pie chart was used to indicate the richness (frequency) of each TcRBV family and each TcRBJ (6 BJ1, 7 BJ2) in each group of the three groups, and the frequencies of TcRBV (BJ) between the three groups were compared respectively; was no significant difference is found (data no shown).

### Comparison of clonal overlap rate of TcRBV CDR3 between groups

The clonal overlap rate between samples in each group was obtained from the profile of immune repertoire (IR) that was derived from HTS and IR alignment. The Baroni–Urbani and Buser (BUB) index was used to indicate the clonal overlap rate between the samples, and there was no significant difference between the three groups (*P* > 0.05, Additional file 1: Tab. S2). The heatmap and boxplot of the overlap rate are shown (Additional file 2: Fig. S3).

### Relationship between CDR3 diversity and immune system stability

**Fig. 3:**
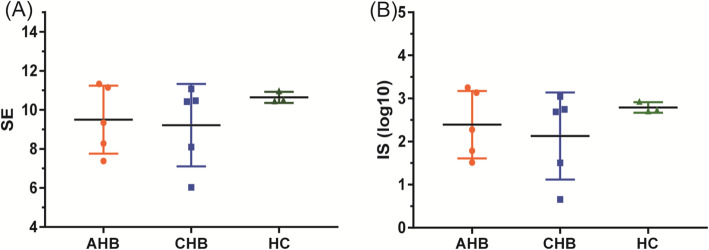
SE and IE of CDR3 diversity. Shannon entropy (SE) index (**a**) and inverse Simpson’s (IE) index (**b**) used as indicators of CDR3 diversity of TRBV in groups

The diversity of V/D/J gene clones in the three groups were compared and analyzed based on the above-mentioned T cell CDR3 immune repertoire (IR). SE and IS of the three groups were compared considering each clone as different species, and the number of each clone sequence as the richness of the species. There was no significant difference in the SE and IS found between the three groups (P > 0.05). The detailed SE and IS are shown in Fig. 3.

## Discussion

T cells play an important role in limiting HBV infection, and their CDR3 of the TCR β chain is synthesized by three genes of the V/D/J segments, through rearrangement of the gene segments [23]. This forms a variety of CDR3 clones that can respond to various antigens. The diversity of CDR3 can regulate immune homeostasis by affecting the cytotoxic activity of effector T cells [24, 25]. HBsAg SC is a sign of good outcome in subjects with asymptomatic or acute HBV infection although the mechanism involved is still unclear [26]. This study analyzed the clone type and diversity of CDR3 immune repertoire (IR) in resolved acute hepatitis B (AHB) subjects with HBsAb positive (HBsAb+), and screened the clone types that would be relate to HBsAg SC in subjects infected with HBV.

Immune repertoire (IR) refers to the sum of all functional diversity B cells and T cells in the circulatory system of an individual at any given time. High throughput sequencing (HTS) technology can truly and comprehensively discover the genetic information of all T-cell receptors (TcR), and studying the CDR3 sequence of all TcRs [27]. HTS could broadly reveal the diversity and complexity of TcR; it is thus an important technology for exploring IR. The adaptive immune system monitors pathogens through the diversity of CDR3 in IR [28].

To study the difference of CDR3 IR among subjects with or without HBsAb positive (HBsAb+), 5 AHB cases (HBsAb+) were compared with 5 CHB cases and 3 healthy controls (HC). The results showed that the clone overlap rate of the AHB group was the highest and that the HC group was the second among the three groups. This higher overlapping rate represents a higher CDR3 homology, which means that the subject with a higher overlapping rate of CDR3 undergoes easier recovery after HBV infection.

In order to further explore the effect of different TcRBV clone types on the HBsAg SC in subjects with HBV infection, we compared the top ten clone types between three groups and obtained the clone types (TcRBV20–1/BD1/BJ1–2) with significant differences between AHB and HC groups. This indicates that the clone types may be related to different outcomes in subjects with HBV infection (AHB or CHB) [29]. Among non-dominant clones, there were 57 TcRBV clone types with a significant difference in AHB compared with that of the CHB group, of which TcRBV6–4/BD1 (BD2) /BJ2–2 had the highest cloning frequency, and a higher frequency was found in the AHB group than in CHB group; the frequency was also higher in the AHB group than that in the HC group, indicating the close association of this genotype with HBsAg SC [30]; however, the number of cases needs be expanded for further verification. Furthermore, whether the different TcRBV genotypes are specific for HBV antigen, should be confirmed in future studies.

Diversity is an indicator to measure the variation among hosts. In the immune system, the higher diversity of immune cells, the higher is their stability and the stronger is their ability to resist the invasion of various antigens (pathogens). Conversely, the host is more susceptible to pathogens and tumors, if the diversity of the immune system is low (that is, smaller number of diverse T cell clones) [31–33]. After HBV infection, some of the infected subjects manifest acute asymptomatic infection and HBsAg SC in peripheral blood, becoming convalescent. Some subjects progress to chronic persistent HBV infection develop chronic hepatitis B (CHB). In addition, the different outcomes of HBV infection may be attributed to the diversity of individual immune status, especially the diversity of T cell clones [5, 25].

HBsAb production is the aim of hepatitis B vaccination. However, 5–10% of recipients is unable to produce a protective level of antibodies against HBsAg after a standard vaccine course [34]. There are few reports about the relationship of the profile of the TcRBV family and the result of HBV vaccination. Whitacre, D. C. et al. have indicated that the clonal change of TcRBV gene in CD4 + T cells is one of the important factors affecting the immune response of the recipients to hepatitis B vaccine [35].

In this study, diversity indices (SE and IS) were used to assess the diversity of T cell clones (TcRBV CDR3), and the results showed no significant difference between the three groups in the CDR3 sequence, suggesting that there was no significant difference in immune status among the three groups. Miyasaka et al. suggested that an increased TCR β chain diversity and a decreased B cell receptor (BCR) IgG H chain diversity after the second HB vaccination could predict a better response to HB vaccination [6, 36], indicating different mechanisms by which the host produces HBsAb after responding to symptomatic HBV infection or HBV vaccination. The results further hint that the no-response of hepatitis B vaccination can be related to the immune status of the body itself [37], though, further studies with additional cases are needed to confirm this possibility. Additionally, several limitations are present in the present study. First, the number of samples tested in each group was small, especially only three subjects in the HC group. We were also unable to compare sex differences among subjects because only male subjects were used in the AHB group [38].

## Conclusions

The results suggest that there are 57 TcRBV clonotypes may be related to the HBsAg SC in AHB subjects, especially the TcRBV6–4/BD1 (BD2) /BJ2–2. Additionally, there may be no significant relationship between the clonal diversity of TCR β chain CDR3 and HBsAg SC. However, further studies with additional subjects are needed to confirm this hypothesis, which will improve the prediction of prognosis of subjects with HBeAg+ CHB.

## Supplementary Information

**Additional file 1: Table S1.** The information of amplification. **Table S2.** Clonal overlap rate (BUB index) among the three groups.

**Additional file 2: Figure S1.** Frequency of V/J gene combinations have significant differences among the three groups (*P* < 0.001). **Figure S2.** Frequency of V/J gene combinations have significant differences among the three groups (0.001 < *P* < 0.05). **Figure S3.** Heatmap of clonal overlap rate of CDR3 for samples in each group.

## Data Availability

The datasets used in the current study are available from the corresponding author on reasonable requirements.

## Abbreviations

AHB: Resolved acute hepatitis B
CDR3: Complementarity determining region 3
HTS: High throughput sequencing
IS: Inverse Simpson’s index
PBMC: Peripheral blood mononuclear cells
PCR: Polymerase chain reaction
RT: Reverse-transcription
SC: Seroconversion
SD: Standard deviation
SE: Shannon entropy index
TcRBV: T-cell receptor beta chain variable region
