# Supplementary material for: Restricted TcR β chain CDR3 clonotype is associated with resolved acute hepatitis B subjects
Source: BMC Infect Dis. 2021 Jan 23;21:111. doi: 10.1186/s12879-021-05816-2 (PMC7825183; doi:10.1186/s12879-021-05816-2)
Supplement: Supplementary file 1 — Additional file 1: Table S1. The information of amplification. Table S2. Clonal overlap rate (BUB index) among the three groups. [file 12879_2021_5816_MOESM1_ESM.docx]

# Additional file 1:

**Table S1** The information of amplification

| Groups | Sample | Total Reads | Unique CDR3 | CDR3 | Unique CDR3/CDR3 | Pielous/Diversity |
| --- | --- | --- | --- | --- | --- | --- |
| AHB | H4 | 6471390 | 71037 | 159817 | 0.4445 | 0.7245 |
|  | H5 | 5597745 | 125886 | 224317 | 0.5612 | 0.8872 |
|  | H6 | 5799381 | 92996 | 127232 | 0.7309 | 0.9149 |
|  | H14 | 5653381 | 176923 | 260945 | 0.6780 | 0.9163 |
|  | H16 | 6971334 | 90239 | 323057 | 0.2793 | 0.5283 |
| CHB | B1 | 5674724 | 53821 | 92741 | 0.5803 | 0.8575 |
|  | B2 | 6632270 | 74724 | 166263 | 0.4494 | 0.7377 |
|  | B6 | 5282228 | 173365 | 253599 | 0.6836 | 0.9393 |
|  | B7 | 5676476 | 89425 | 330128 | 0.2709 | 0.6474 |
|  | B9 | 5056708 | 160941 | 274563 | 0.5862 | 0.9298 |
| HC | H3 | 5470001 | 74724 | 97021 | 0.7702 | 0.9317 |
|  | H9 | 5166218 | 183700 | 286885 | 0.6403 | 0.9049 |
|  | H18 | 5370926 | 87632 | 129930 | 0.6745 | 0.9230 |

Note: Merged clonal reads from the same template by using Unique Molecular Barcode (UMB)

## Table S2 Clonal overlap rate (BUB index) among the three groups

| Groups | Overlap rate Median (min~max) | *P* |
| --- | --- | --- |
| AHB | 2.00% (1.74%~2.30%) | 0.2521^a^ |
| CHB | 1.77% (1.43%~2.61%) | 0.8836^b^ |
| HC | 1.82% (1.62%~2.12%) | 0.4968^c^ |

^a^ *P*, AHB vs. CHB; ^b^*P*, CHB vs. HC; ^c^*P*, AHB vs. HC
