# Supplementary material for: Restricted TcR β chain CDR3 clonotype is associated with resolved acute hepatitis B subjects
Source: BMC Infect Dis. 2021 Jan 23;21:111. doi: 10.1186/s12879-021-05816-2 (PMC7825183; doi:10.1186/s12879-021-05816-2)

# Additional file 2:

## Figure S1

Frequency of V/J gene combinations have significant differences among the three groups (P <0.001). (a) AHB & CHB; (b) AHB & HC; (c) CHB & HC


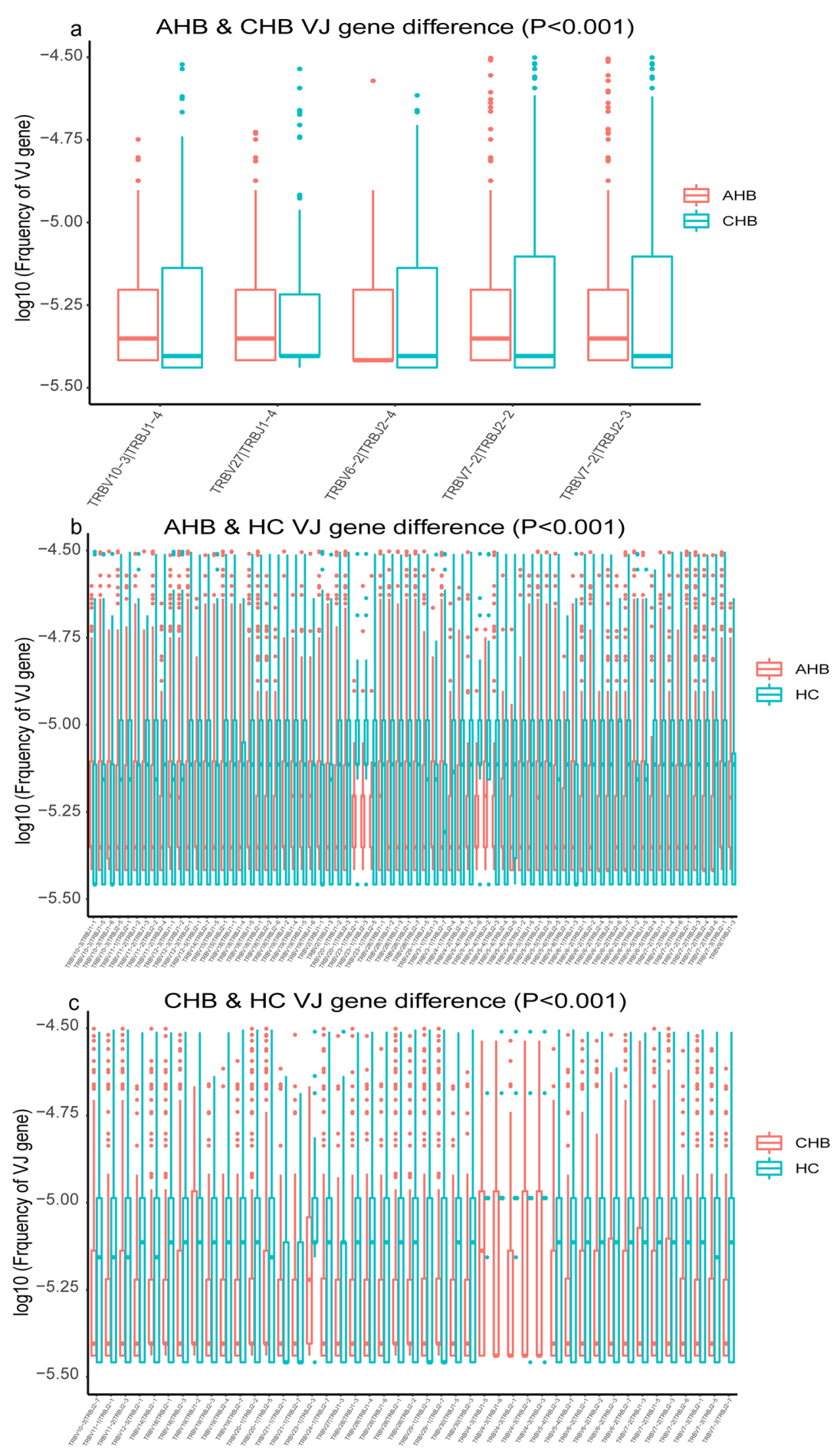


## Figure S2

Frequency of V/J gene combinations have significant differences among the three groups (0.001<P < 0.05). (a) AHB & CHB; (b) AHB & HC; (c) CHB & HC


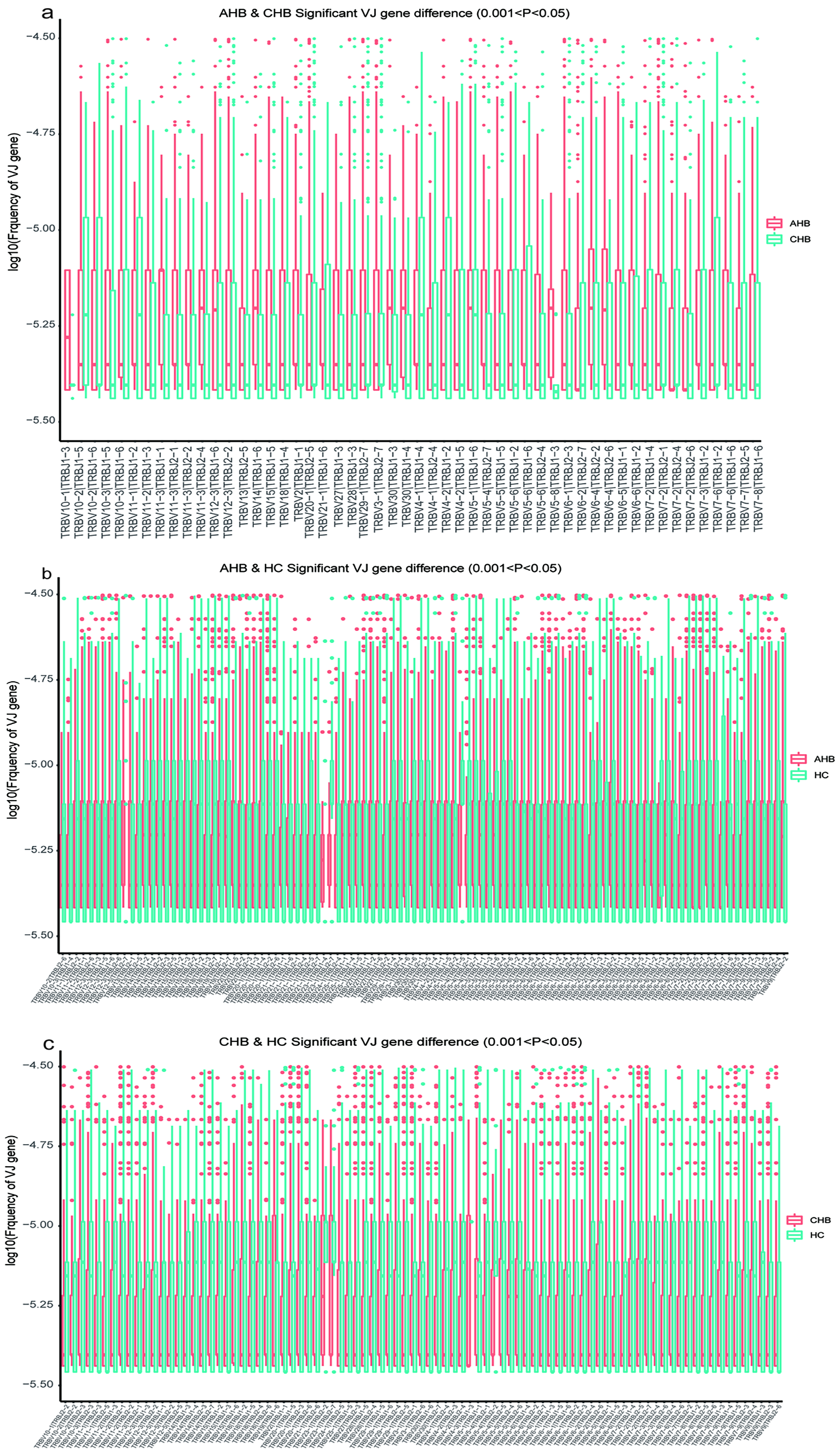


## Figure S3

## Heatmap of clonal overlap rate of CDR3 for samples in each group.

BUB index between samples in the groups AHB (A), CHB (B), and HC (C), there is no significant difference of BUB index between the three groups (D, *P* > 0.05)


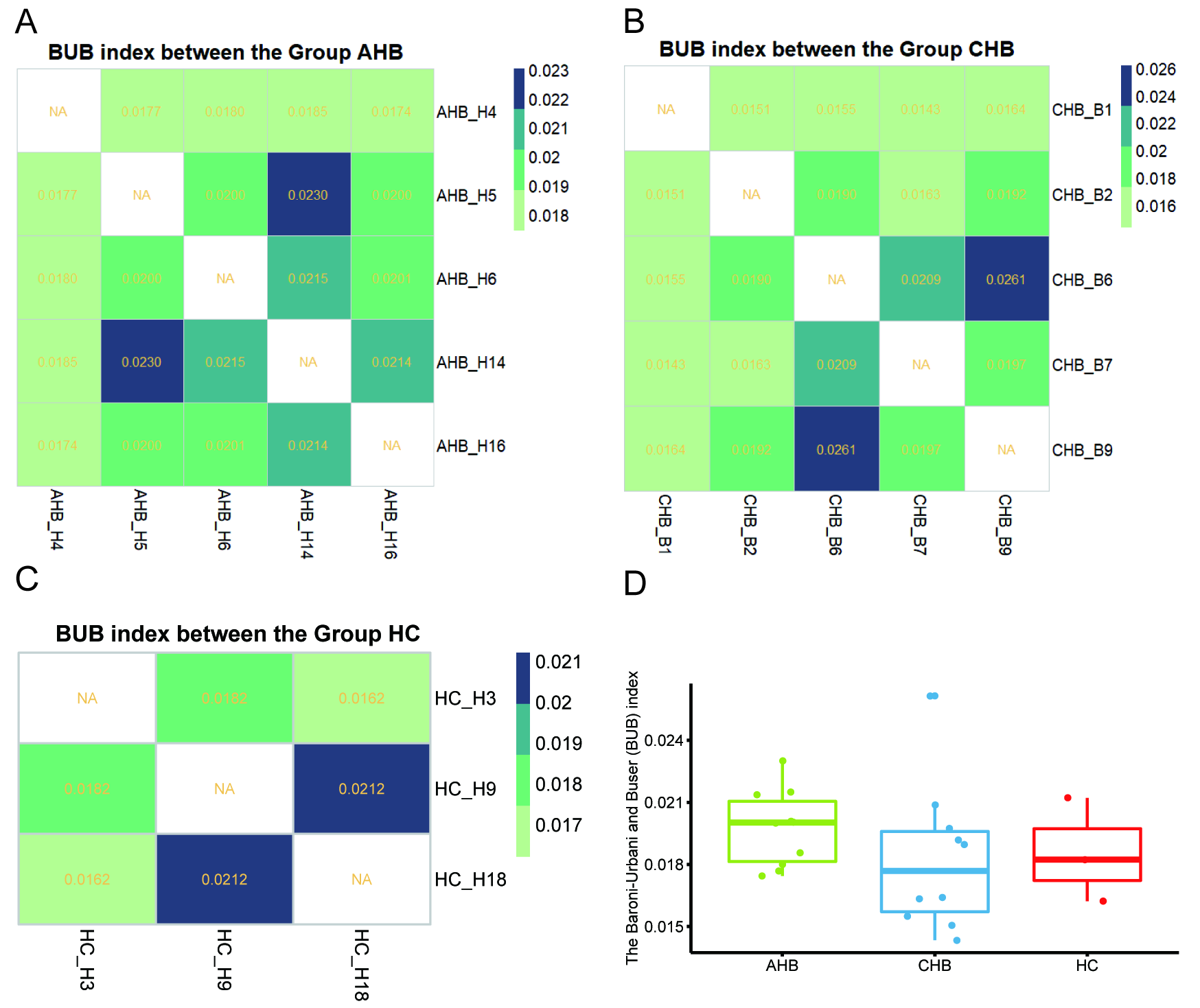

Supplement: Supplementary file 2 — Additional file 2: Figure S1. Frequency of V/J gene combinations have significant differences among the three groups (P < 0.001). Figure S2. Frequency of V/J gene combinations have significant differences among the three groups (0.001 < P < 0.05). Figure S3. Heatmap of clonal overlap rate of CDR3 for samples in each group. [file 12879_2021_5816_MOESM2_ESM.docx]
